# Progress in Adaptive Immunotherapy for Cancer in Companion Animals: Success on the Path to a Cure

**DOI:** 10.3390/vetsci2040363

**Published:** 2015-10-19

**Authors:** Katie L. Anderson, Jaime F. Modiano

**Affiliations:** 1 DVM/PhD Combined Degree and Comparative Molecular Biosciences Graduate Programs, Department of Veterinary Clinical Sciences, College of Veterinary Medicine and Animal Cancer Care and Research Center, University of Minnesota, St. Paul, MN 55108, USA; 2 Department of Veterinary Clinical Sciences, College of Veterinary Medicine, Animal Cancer Care and Research Center, Center for Immunology, Masonic Cancer Center, and Stem Cell Institute, University of Minnesota, Minneapolis, MN 55108, USA; E-Mail: modiano@umn.edu

**Keywords:** adaptive immunity, T-lymphocytes, antibodies, immunization, Fas ligand, virotherapy

## Abstract

Harnessing the ability of the immune system to eradicate cancer has been a long-held goal of oncology. Work from the last two decades has finally brought immunotherapy into the forefront for cancer treatment, with demonstrable clinical success for aggressive tumors where other therapies had failed. In this review, we will discuss a range of therapies that are in different stages of clinical or preclinical development for companion animals with cancer, and which share the common objective of eliciting adaptive, anti-tumor immune responses. Even though challenges remain, manipulating the immune system holds significant promise to create durable responses and improve outcomes in companion animals with cancer. Furthermore, what we learn from this process will inform and accelerate development of comparable therapies for human cancer patients.

## 1. Introduction

The field of cancer immunotherapy, which seeks to harness and enhance the ability of the immune system to eliminate cancer, has gained considerable interest in recent decades with the success of immunotherapeutics in clinical trials for human patients with a variety of hematopoietic and solid tumors [1,2,3,4]. The field of veterinary immunotherapy holds similar promise for companion animals with cancer. The immune system can uniquely target cancer cells or the tumor microenvironment while minimizing damage to normal tissues, and immune cells can reach surgically inaccessible locations within the body [3,4]. Additionally, immunological memory may provide long lasting, durable clinical responses [3,4].

Both passive and active modalities have been used to generate therapeutic anti-tumor immune responses. Passive immunotherapy involves the transfer of biological reagents, such as monoclonal antibodies or antigen-specific adaptive immune cells, into the cancer patient [3]. Active immunotherapy seeks to elicit an anti-tumor response from the patient’s own immune system, typically through vaccination [3]. In this review, we will focus on strategies that strive to activate the adaptive immune system. An effective anti-tumor adaptive immune response requires the processing and presentation of tumor-associated antigens (TAAs) by antigen presenting cells to T cells followed by T cell activation and proliferation [4]. The primary obstacle opposing this response is immune tolerance [4,5]. Cancer cells evolve under the selective pressures of immuno-editing, and the resulting tumor may present very few TAAs recognizable to T cells [4,5]. In addition, the tumor microenvironment evolves to create a highly immunosuppressive barrier that limits the effectiveness of an immune response [4]. Successful immunotherapies seek to circumvent or mitigate immune tolerance to reestablish anti-tumor immunity.

Comparative oncology and comparative immunology have the potential to not only improve the lives of companion animals with cancer, but also to significantly inform human clinical trials. Client-owned dogs and cats develop spontaneous malignancies with similar etiologies, such as genetic abnormalities and common environmental exposures, to human cancers [5]. Immunocompetent dogs and cats represent a more outbred population than laboratory mice and thus are likely to represent a wider spectrum within the heterogeneity of responses to immunotherapy, such as are seen in human populations. Our current understanding of the anti-tumor immune response and of the effects of immunotherapy is incomplete. As it is often easier to obtain clinical samples from our veterinary patients than from human subjects, studying the similarities and differences in the human and dog immune response may provide the insights needed to optimize immunotherapies. Veterinary trials also represent opportunities to develop improved therapeutic modalities, optimize dosing schedules, and identify biomarkers to predict and identify patient responses.

## 2. Passive Immunotherapy: Monoclonal Antibodies

Since the 1997 FDA approval of rituximab, a mAb designed to target the B-cell marker CD20, a large panel of mAbs has been approved for the treatment of both hematologic and solid malignancies [6]. mAbs have quickly become a mainstay of cancer treatment by consistently increasing patient remissions with minimal toxicity [6]. Initially, mAb therapy was impeded by their production in mice and subsequent immunogenicity in humans [7]. Today, a number of techniques are used to “humanize” murine antibodies for clinical use by grafting the mouse complementarity-determining region onto a recombinant human immunoglobulin backbone [7]. The development of technology to speciate antibodies has led to a variety of clinical trials in companion dogs [8]. New forms of mAbs, such as bispecific antibodies and immunoconjugates, are also being developed and show promise in initial clinical trials.

**Figure 1:**
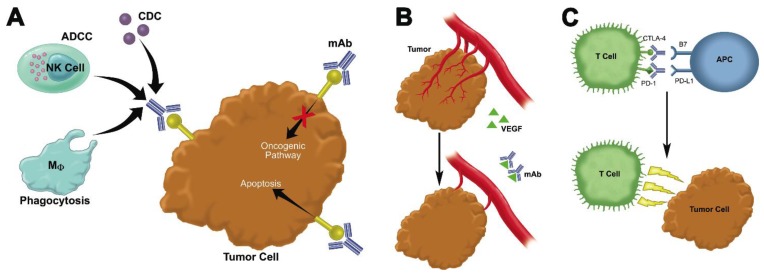
Mechanisms of targeting tumors using monoclonal antibodies (**A**) The first class of mAbs binds directly to tumor cells and induces apoptosis or antagonizes oncogenic pathways. The Fc region of the mAb may also induce antibody-dependent cellular cytotoxicity (ADCC), complement-dependent cytotoxicity (CDC) or phagocytosis of the tumor cell. (**B**) The second class of mAbs blocks growth promoting pathways in the stroma. In the example shown here, an anti-VEGF mAb acts to prevent angiogenesis. (**C**) The third class of mAbs, immune checkpoint inhibitors, blocks the interaction of inhibitory receptors expressed on activated T cells (CTLA-4, PD-1) with ligands on antigen presenting cells (B7, PD-L1) or tumor cells (PD-L1 or PD-L2). This therapy prevents the attenuation of the T cell response and allows activated T cells to kill tumor cells.

mAbs are designed to modulate targets expressed on the surface of cancer cells or in the tumor microenvironment [7]. Three main classes of mAbs are currently in use (Figure 1): (1) mAbs that directly bind to malignant cells and antagonize oncogenic pathways; (2) mAbs that act to block growth-promoting pathways in the tumor stroma; and (3) mAbs, termed immune checkpoint inhibitors, which act to directly modulate the activity of anti-tumor adaptive immune cells [6]. In addition to their primary effects, mAbs mediate an anti-tumor immune response via the interaction of the Fc portion of the antibody with the corresponding receptor on immune effector cells. This interaction triggers antibody-dependent cellular cytotoxicity, phagocytosis, and complement-dependent cytotoxicity and may lead to antigen presentation and the generation of an adaptive immune response [4]. Ongoing studies aim to further enhance the interaction of mAbs with immune cells by modulation of the antibody structure [4].

### 2.1. Monoclonal Antibodies That Bind to Malignant Cells and Antagonize Oncogenic Pathways

The first mAbs to demonstrate therapeutic efficacy targeted antigens expressed on the surface of cancer cells (Figure 1A) [7]. For hematologic malignancies, anti-CD20 (Rituximab) has revolutionized the treatment of B-cell lymphoma and increased the rate of durable remissions from 30% to 60% [9]. In addition to stimulating innate immunity through the Fc receptor, anti-CD20 acts by directly inducing tumor cell apoptosis. Recent studies have suggested that this tumor cell destruction results in the cross-presentation of tumor antigens and the stimulation of a durable anti-tumor adaptive immune response [10,11]. The consistent expression of CD20 has been confirmed in canine B cell lymphoma, and chimeric anti-CD20 antibodies speciated to dog have been developed [9,12,13,14,15]. Interestingly, although the proposed rituximab binding epitopes are conserved between human and canine CD20, the canine mAb does not induce direct apoptosis of tumor cells. Studying the differences in the mechanism of action between human and canine anti-CD20 mAbs may yield insights to improve the engineering of mAbs. Despite these differences, each of the canine anti-CD20 mAbs has been shown to have diagnostic or clinical potential. For example, *in vitro*, 6C8 anti-canine CD20 mAb labeled 100% of canine B-cell lymphomas tested, enhanced antibody dependent cellular cytotoxicity, and promoted macrophage phagocytosis of tumor cells [9]. Similarly, *in vivo* studies showed reduced tumor burden in mice harboring canine CLBL1 B cell lymphoma xenografts after treatment with 1E4 anti-canine CD20 antibody monotherapy [12]. In a prospective, randomized clinical trial, treatment with AT-004 anti-canine CD20 was reported to increase median progression-free survival of dogs with B cell lymphoma [16]. In addition to anti-CD20, another monoclonal antibody, anti-CD47, has been shown to improve the innate anti-tumor immune response in both *in vitro* and *in vivo* xenograft models of several human leukemias and lymphomas [17,18]. Preliminary data indicate that these therapeutic properties of anti-CD47 are retained in the setting of canine B-cell lymphoma (Modiano *et al.*, manuscript in preparation). mAb therapy for the treatment of T cell lymphoma has also been developed. AT-005 (Aratana Therapeutics, Del Mar, CA, USA), a speciated mAb targeting CD52 on T cells, has received conditional approval from the USDA and is currently being tested in canine clinical trials [19].

mAbs used in the treatment of solid malignancies often antagonize oncogenic receptor tyrosine kinases to reduce proliferative signaling (Figure 1A) [7]. Therapeutically efficacious antibodies include anti-EGFR (Cetuximab) and anti-HER2 (Trastuzumab) used in various epithelial cancers and in HER-2-overexpressing breast cancer, respectively [4,7]. In murine models, both antibodies have been shown to enhance priming of anti-tumor CD8+ T cells and increase pro-inflammatory cytokine release; however, in human patients treated with anti-EGFR, the frequency of intratumoral, immunosuppressive Tregs was increased [20]. Thus the effect of these antibodies on the adaptive immune response is yet unknown. Veterinary trials have the potential to help elucidate this mechanism of action and identify strategies to improve the anti-tumor immune response. Both EGFR and HER2 have structural homologs in various canine cancers, and chimeric versions of these antibodies speciated to the dog have been developed [8]. Canine anti-EGFR was shown to decrease canine mammary carcinoma cell proliferation by 40%–60% and to mediate tumor cell killing by macrophage phagocytosis *in vitro* [8]*.* Overexpression of HER2 has also been identified in spontaneous feline mammary carcinomas; however, feline specific antibodies have yet to be developed [21,22]. Ongoing and future clinical studies will continue to evaluate the *in vivo* efficacy of these reagents and their effects on the adaptive immune response.

### 2.2. Monoclonal Antibodies That Block Growth-Promoting Pathways in the Tumor Stroma

A second class of mAbs acts to neutralize the growth-promoting effects of the tumor microenvironment (Figure 1B) [6]. For example, bevacizumab, a humanized mAb against vascular endothelial growth factor (VEGF), has been shown to have anti-angiogenic effects in a variety of human cancers [23,24]. Recent murine studies also have suggested that low doses of anti-angiogenic mAbs serve to “normalize” the tumor vasculature, which subsequently improves the infiltration of effector T cells into the tumor and reprograms the hypoxia-induced immunosuppressive microenvironment [25,26]. These results suggest that bevacizumab may synergize with other immunotherapies, and veterinary clinical trials may provide the opportunity to develop efficacious combination treatment schedules. In a mouse xenograft model of canine hemangiopericytoma, bevacizumab treatment suppressed tumor growth by inhibiting angiogenesis [23]. Similarly, *in vivo* studies have shown that mice harboring canine osteosarcoma xenografts had significantly delayed tumor growth when treated with either high dose or low dose bevacizumab as compared to a control [24]. These studies demonstrate that anti-angiogenic mAbs may be therapeutically efficacious in inhibiting the growth of canine sarcomas.

### 2.3. Immune Checkpoint Inhibitors

The third and most recently developed class of mAbs, termed immune checkpoint inhibitors, has generated considerable interest in the field of immunotherapy by demonstrating the ability to induce durable clinical responses in a subset of patients [27,28,29,30]. Immune checkpoint molecules, such as CTLA-4 and PD-1, act to limit the efficacy of the anti-tumor response by inducing anergy or exhaustion in activated T cells [27,30]. Antibodies against CTLA-4, PD-1, and its corresponding ligand PD-L1 aim to reactivate tumor-specific T cells and cause a robust anti-tumor immune response (Figure 1C) [27,29]. In human phase-III clinical trials, checkpoint inhibitors induced responses in 20%–65% of patients with a variety of tumor types; a small percentage of these patients have achieved complete, durable remissions lasting several years [29]. Although checkpoint inhibitors have yet to be tested in canine clinical trials, expression of canine PD-L1 has been detected on a number of canine tumor types, including mastocytoma, melanoma, renal cell carcinoma, and several others [31]. Treatment of canine tumor infiltrating lymphocytes with anti-PD-L1 enhanced IFN-γ production, suggesting that blockade with this antibody may provide therapeutic benefit for dogs harboring PD-L1^+^ tumors [31]. Canine CTLA-4 has also been identified and cloned [32]. While canine anti-CTLA-4 has not yet been developed, an agonistic recombinant canine CTLA-4 molecule has been successfully used to induce tolerance in a transplant model [33]. This demonstrates that the mechanism of action of CTLA-4 is conserved between humans and dogs, and CTLA-4 blockade could be clinically efficacious in canine cancer.

### 2.4. Bispecifics, Trispecifics, Immunoconjugates, and Other Modified Antibodies That Enhance the Interaction between Immune Cells, Tumor Targets, and the Tumor Microenvironment

Ongoing work aims to enhance the therapeutic efficacy of mAbs using antibody engineering [6,34]. Bispecific or trispecific antibodies possess affinity for two or three different antigens and may or may not retain their ability to activate the innate immune system through the Fc region, depending on their design [34]. These mAbs may be used to crosslink two distinct tumor antigens to block oncogenic signaling pathways [34]. Alternatively, bispecific mAbs may be used to recruit immune effector cells and place them in close proximity with tumor cells [34]. Bispecific T cell engagers (BiTEs) consist of two single chain variable fragments (scFv) connected by a linker: one binds to a tumor antigen and one binds to CD3 on T cells [35]. Several BiTEs are currently in clinical trials, including Blinatumomab, a BiTE directed against CD19 for the treatment of ALL [34,35]. Similarly, bispecific killer engagers (BiKEs) contain a scFv against CD16 to recruit natural killer (NK) cells as well as a scFv against a tumor antigen [36]. Although these technologies have yet to be applied to veterinary medicine, bispecific mAbs represent a powerful tool for future clinical use.

Antibody engineering technology has also been used to create immunoconjugates: antibodies designed to provide targeted delivery of cytotoxic drugs or radioactive isotopes to tumor cells [6,37]. Antibody drug conjugates (ADCs) are composed of a targeted mAb stably linked to a cytotoxin, which is released following the internalization of the compound by a tumor cell. This targeted delivery limits systemic exposure and results in fewer side effects and a wider therapeutic window [38]. Currently two ADCs, brentuximab vedotin (Adcetris^®^, Seattle Genetics, directed against the CD30 antigen expressed in some lymphocytes) and trastuzumab emtansine (Kadcyla^®^, Genentech, targeted against the HER2/neu antigen that is overexpressed by some breast cancers) are approved for use in refractory Hodgkin’s lymphoma and metastatic breast cancer, respectively, and more than 30 other compounds are being tested in human clinical trials [38]. Another application of this technology is in the use of radioimmunotherapy to condition the bone marrow niche prior to nonmyeloablative hematopoietic stem cell transplantation [39,40,41]. The canine model has been utilized to establish the effective dose of anti-CD45 and anti-TCRαβ mAbs conjugated to bismuth-213 [39] and anti-CD45 conjugated to Astatine-211 [41] to induce myelosuppression with minimal off target toxicity. Successful hematopoietic stem cell transplants were achieved in both studies, demonstrating the efficacy of using radioimmunotherapy as part of a conditioning regime [39,41].

Although mAbs were first developed several decades ago, their clinical success in humans has driven continuing advances in antibody engineering and resulted in the development of a diverse array of therapeutics. The success of mAbs such as Rituximab, Cetuximab, and checkpoint blockade inhibitors in human medicine strongly suggests that these therapeutics are likely to succeed in veterinary medicine as well. Despite their potential efficacy, few speciated antibodies have been developed for veterinary medical use or tested in veterinary clinical trials. mAbs have the ability to target a variety of both hematopoietic and solid tumors and can be used “off the shelf”, *i.e.*, these therapies do not need to be personalized for each individual patient. Antibody manufacturing techniques are well established, so cost is not likely to be a single major obstacle that would prevent their implementation. Therefore, mAb therapy represents one of the most promising avenues for the development of veterinary immunotherapy. In the following sections, we will discuss active immunotherapy techniques that, although they are less established than mAb therapy, hold additional promise for durable clinical responses.

## 3. Active Immunotherapy: *In Situ* Immunization with Adenovirus-Fas Ligand

**Figure 2:**
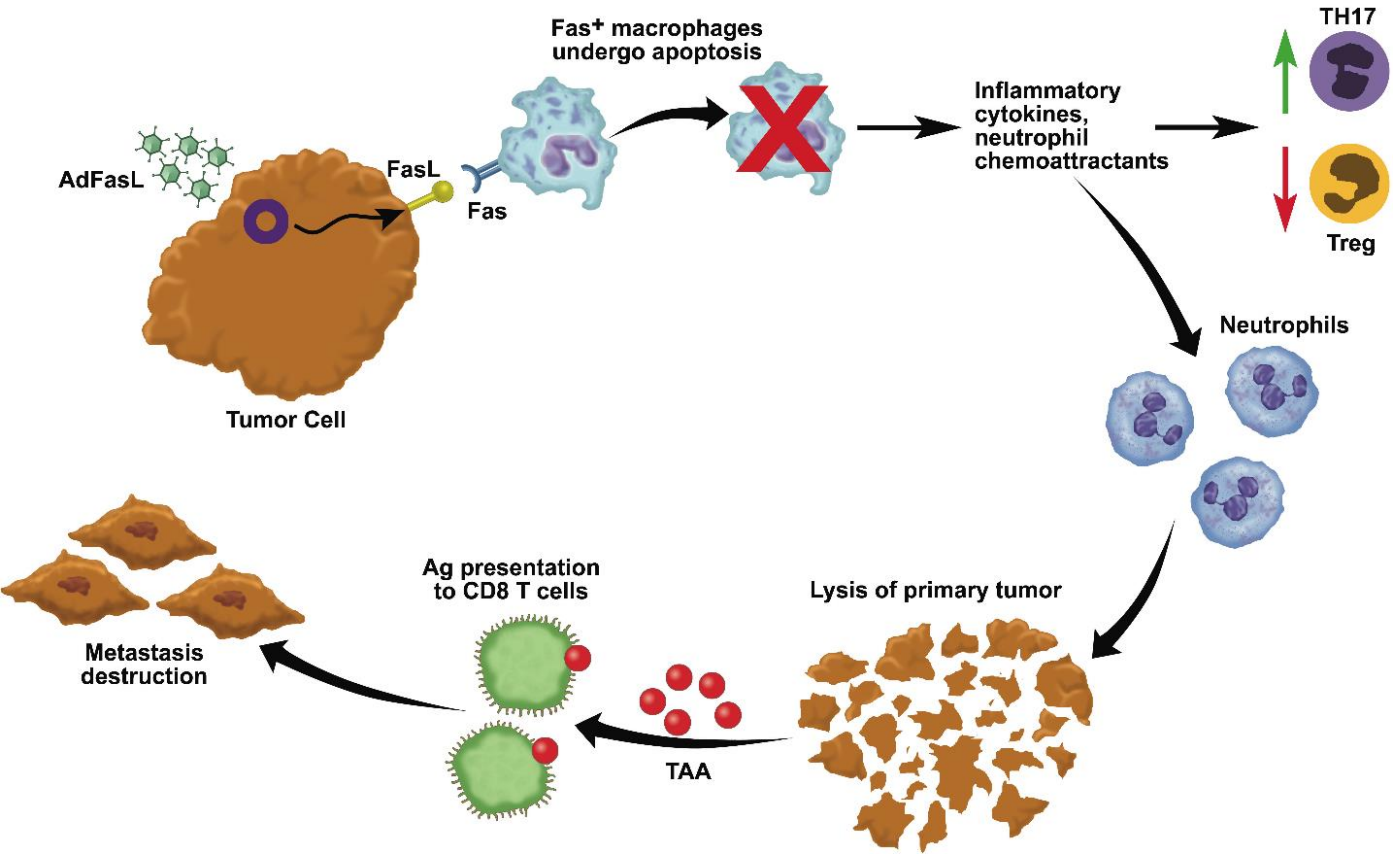
AdFasL induces an anti-tumor inflammatory response in the tumor microenvironment. Interaction with FasL+ tumor cells causes macrophages to undergo apoptosis and release inflammatory cytokines and neutrophil chemoattractants. The infiltrating neutrophils are responsible for localized tumor cell destruction and the release of tumor associated antigens (TAA). These antigens are cross-presented to cytolytic CD8 T cells, which locate and destroy tumor cells at distant metastatic sites and provide protection from further challenge via immunologic memory. In addition, cytokines released by macrophages drive the differentiation of pro-inflammatory Th17 T cells [50] and induce apoptosis of anti-inflammatory T regulatory cells.

Stimulation of an anti-tumor immune response can be achieved through *in situ* immunization strategies that promote inflammation and necrosis at the primary tumor site (Figure 2) [42]. Intratumoral FasL gene therapy represents one such strategy. The interaction of the Fas “death receptor” with its ligand (FasL) triggers an apoptotic death pathway through the recruitment of the Fas-associated death domain and pro-caspase 8 [43]. Ectopic expression of FasL leads to the rapid rejection of tumors in mouse xenograft models [44,45] and induces anti-tumor immune responses that protect mice from subsequent tumor challenge [42]. This therapy has been used to treat a variety of syngeneic and xenotransplanted tumors in the pre-clinical setting, including prostate cancer, lung cancer, colon cancer, lymphoma, melanoma, and neuroblastoma in mice, and melanoma and osteosarcoma in dogs reviewed in ref. [42], highlighting its potential value for treatment of multiple types of human cancers.

Mechanistically, in Fas-sensitive tumors, FasL administration leads to apoptotic cell death [42]. However, tumor rejection does not require expression of Fas by the tumor cells; instead, it is the expression of Fas in the tumor microenvironment which leads to an anti-tumor inflammatory response [43]. After interacting with FasL+ tumor cells, macrophages undergo apoptosis and release significant levels of inflammatory cytokines and neutrophil chemoattractants [46]. The infiltrating neutrophils are responsible for localized tumor cell destruction [45,47,48] and the release of tumor antigens [47,49]. These antigens are cross-presented to cytolytic T cells, which locate and destroy tumor cells at distant metastatic sites and provide protection from further challenge via immunologic memory [47,49]. In addition, cytokines released by macrophages, including IL-1β, IL-6, TGF-β, and IL-23 drive the differentiation of pro-inflammatory Th17 T cells [50] and induce apoptosis of anti-inflammatory T regulatory cells to potentiate the anti-tumor immune response [51]. In summary, when administered intratumorally, FasL induces massive suppurative inflammation, local destruction of the primary tumor, and a durable adaptive immune response.

Gene transfer of FasL can be safely and effectively achieved *in vivo* using an adenovirus vector (AdFasL). This approach induces supraphysiologic FasL expression in both tumor cells and cells in the local microenvironment to enhance therapeutic efficacy [52,53]. In addition, the potential for systemic or chronic toxicity is reduced by the self-limiting nature of AdFasL therapy: attrition of the adenovirus and death of the infected cells extinguishes the FasL-dependent response [42]. Administration of FasL was initially tested in the clinical setting by intratumoral administration of naked plasmid DNA in five dogs with oral malignant melanoma [52]. This study showed that FasL administration was safe and reduced local tumor burden, and in four of five subjects treated, it was associated with objective responses [52]. *In situ* immunization with FasL was subsequently tested using a replication-deficient adenovirus platform in a clinical study of 56 dogs with spontaneous osteosarcoma in the neoadjuvant to standard of care setting [43]. This trial confirmed the safety of AdFasL with no significant therapy-related adverse events [43]. Predictably, tumors with reduced Fas expression showed increased levels of local inflammation, necrosis, and lymphocytic infiltration upon treatment with AdFasL. Dogs with elevated inflammation scores (>2–3) also demonstrated a 65% increase in median survival time compared to untreated controls (359 *vs.* 221 days) [43]. Responses to AdFasL were also durable: 38% of the treated dogs were alive at one year compared to 20% of dogs receiving the standard of care alone [43]. Tumors with elevated Fas expression developed lower levels of inflammation (score ≤1), and the median survival time of these dogs did not differ from that of untreated controls [43]. Overall, this study suggests that AdFasL gene therapy improved outcomes in a subset of dogs with cancer, specifically those harboring tumors that are resistant to Fas-mediated death signals, making the therapy amenable to patient selection through companion diagnostic tests. Ongoing work seeks to further translate this therapy for applications to treat a variety of solid tumors in humans and companion animals.

## 4. Administration of Attenuated Bacteria

The use of attenuated bacteria to stimulate an anti-tumor immune response has been employed by physicians for over two centuries, most notably by Dr. William Coley in the early 19th century [54]. Although previous therapies were developed as non-specific stimulants of the innate immune system, current work in this field is focused on developing more specific immunotherapies that activate the adaptive immune response [54]. Genetically modified facultative anaerobic bacteria, such as *Salmonella typhimurium* and *Listeria monocytogenes*, display tropism for hypoxic tumor tissue and can be used to induce tumor cytotoxicity, to disrupt the tumor microenvironment, and to stimulate an anti-tumor immune response [55]. The host response to infection begins with the recognition of bacterial pathogen-associated molecular patterns by toll-like receptors (TLR) on innate immune cells [54]. The activation of TLRs results in the production of pro-inflammatory cytokines and phagocytosis of the bacteria [54]. Once in the phagolysosome, bacterial antigens are processed and presented to CD4+ T cells on MHC class II [54]. *L. monocytogenes* may alternatively escape the phagolysosome into the cytosol, where it may be processed and presented to CD8+ T cells on MHC class I [54]. As such, these bacteria hold the added benefit of inducing a memory immune response. In addition to stimulating immunity, attenuated bacteria serve as vectors for gene delivery and have been engineered to express transgenes encoding for anti-tumor “cytokines, antiangiogenic factors, enzymes, and immunogens” [55]. One final benefit of this therapy is the ability to control these agents with antibiotics in the case of therapy-related adverse events [55].

Currently, several genetically modified strains of *S. typhimurium* and *L. monocytogenes* are being tested in human and veterinary clinical trials for a variety of solid tumors. In a phase-I clinical trial at the University of Wisconsin, an attenuated *S. typhimurium* strain was administered intravenously to 41 dogs with a variety of spontaneous malignancies at varying doses. Bacteria were subsequently cultured from 42% of tumors. Although one study related death occurred, four dogs achieved a complete response, two dogs achieved a partial response, and 10% of the dogs achieved disease stabilization, demonstrating the therapeutic potential of this strategy [55]. A recently completed clinical trial for canine osteosarcoma at the University of Minnesota also utilized an attenuated *S. typhimurim* that has been rendered avirulent but remains highly immunogenic [56]. In these genetically modified bacteria, the genes encoding for replication have been replaced with a truncated IL-2 gene, allowing only the bacteria that produce this immune cell attractant to survive and proliferate [56]. An oral formulation was developed to reduce cytokine-driven systemic toxicity observed with intravenous administration of *S. typhimurium* organisms [55] and efficacy was confirmed in mouse models [57]. When administered orally to 19 dogs in an adjuvant setting, this therapy improved the disease free interval, particularly in dogs with highly aggressive tumors, with no toxicity [58]. Finally, a phase-I clinical trial at the University of Pennsylvania investigated the use of an attenuated *L. monocytogenes* vector engineered to express a chimeric human HER2/neu fusion protein. The agent was administered to 17 dogs with appendicular osteosarcoma and prevented the development of metastasis while increasing overall survival compared to a historical control [59]. The development of attenuated bacterial therapy is still in the early stages and has not yet been well established in human medicine. Although veterinary clinical trials have demonstrated therapeutic benefits, most of the trials have included limited, non-randomized patient populations and further testing is needed to demonstrate efficacy. In the next section, we will discuss oncolytic virotherapy, a therapy that also utilizes attenuated pathogens to stimulate an anti-tumor response but is more established than the use of attenuated bacteria.

## 5. Oncolytic Virotherapy

Oncolytic viruses (OV), which preferentially infect and lyse cancer cells, have shown considerable promise in human, canine, and feline clinical trials [60,61,62]. To date, multiple types of viruses have been tested for their oncolytic abilities, including adenoviruses, morbiliviruses, reoviruses, and poxviruses [62]. When selecting a viral vector, both the safety profile of the virus and the presence of virus-neutralizing antibodies must be considered; for instance, although canine distemper virus shows promise as an OV vector, most dogs have been vaccinated against CDV, which may preclude its use in clinical trials [62]. The advent of genetic engineering has allowed researchers to enhance virus tropism for tumor cells and to enhance a virus’s cytotoxic potential by encoding genes that convert pro-drugs to lethal agents, produce lethal proteins, or enhance the anti-tumor immune response. No OV is currently licensed for human or veterinary use in the United States, although several OVs are being tested in phase-III clinical trials [60]. In 2005, China approved the use of a recombinant adenovirus (Oncorine; Shanghai Sunway Biotech, Shanghai, China) in combination with chemotherapy for head and neck human cancer patients [61,63]. In a phase-III trial, Oncorine increased objective response rates by nearly 40%, although survival data were not published [63].

Oncolytic viruses were originally designed to induce “acute tumor debulking” following direct lysis of the tumor cells [61]. It is now thought that the subsequent inflammation and release of tumor antigens stimulates a host anti-tumor immune response that may be more efficacious in tumor clearance than the initial lysis events [61]. Xenograft models have demonstrated an increase in tumor infiltrating neutrophils, macrophages, and natural killer cells as well as their respective cytokines following intratumoral injection of OVs [62]. Human clinical trials have demonstrated an increased anti-tumor T cell response and an increase in antitumor antibodies following treatment [61]. In addition to eliminating the primary tumor, the engagement of the adaptive immune response may eliminate metastasis and induce durable remissions due to the development of memory cells [62]. A number of OVs, including vesicular stomatitis virus and vaccinia virus, have also displayed anti-angiogenic properties in mouse models by inducing the lysis of infected tumor vascular cells [62,64].

A number of studies have been published examining the therapeutic efficacy of OVs in canine and feline cancer cells *in vitro* and in xenograft models of mice harboring canine tumors, which have been extensively reviewed elsewhere (see ref. [62]). These include several poxviruses and vaccinia viruses that demonstrated considerable anti-tumor activity in xenograft models of canine mammary adenocarcinoma and soft-tissue sarcoma and are currently entering clinical trials [62]. To date, several veterinary clinical trials of OVs have been completed. Intratumoral injection of a canine adenovirus genetically modified to express CD40L led to complete tumor rejection in 5 of 19 canine melanoma patients with no significant toxicity [65]. An adenoviral vector encoding the pro-inflammatory cytokine IL-12 under the control of a heat-inducible promoter has been tested as an adjuvant to radiation therapy in a phase-I clinical trial of cats with soft-tissue sarcomas [66]. Although response rates were not published, the trial established the maximum tolerated dose, demonstrated elevated intratumoral IL-12 levels following OV therapy, and formed the foundation for future clinical trials [66]. An additional trial for cats with soft-tissue sarcomas utilized intratumoral injection of either a canarypox viral vector or an attenuated vaccinia viral vector engineered to express feline or human IL-2 following surgery and radiation [67]. OV treatment significantly reduced the rate of tumor recurrence, with 61% of control animals experiencing recurrence as compared to 39% and 28% in the vaccinia and canarypox groups, respectively [67]. Finally, the maximum tolerated dose of a vesicular stomatitis virus engineered to express IFN-β and the sodium-iodide symporter (NIS) has been established in purpose-bred dogs by researchers at the Mayo Clinic [68] and will likely enter canine clinical trials in the near future.

Although oncolytic virotherapy has reached phase-III clinical trials in human patients, most veterinary trials to date have been performed *in vitro* or in small phase-I clinical trials. Several challenges remain to be overcome, including optimization of viral delivery and dissemination throughout the tumor. In addition, the therapeutic use of attenuated pathogens raises biosafety concerns. Additional risk assessments need to be performed prior to regulatory approval of these therapeutics, which may delay the use of oncolytic viruses as mainstream therapy. Despite the challenges, the studies described here show that oncolytic viruses, as least partly through activation of anti-tumor immunity, have the potential to be clinically efficacious in a veterinary setting.

## 6. Anti-Cancer Vaccines

**Figure 3:**
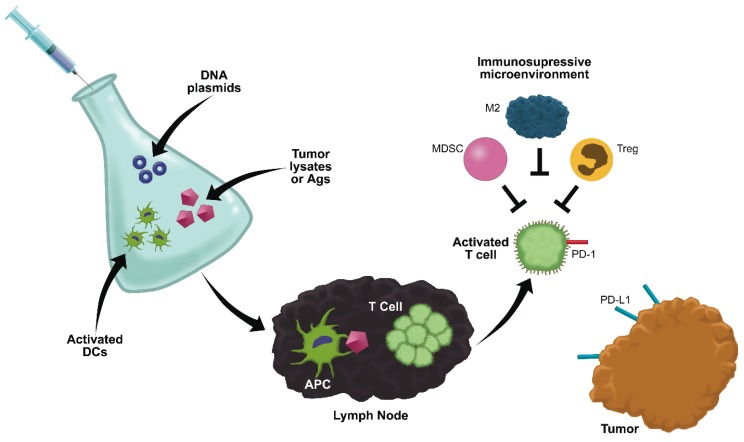
Vaccination strategies and challenges. Vaccines utilize a variety of strategies to activate the immune system against tumor associated antigens (TAAs), including tumor cell lysates or peptide antigens, dendritic cells (DCs) activated with TAAs, and DNA plasmids designed to produce TAAs. The TAAs must then be presented by functional antigen presenting cells to T cells capable of recognizing the TAA. Once activated, T cells must traffic to the tumor and induce tumor cell death. T cell tolerance to TAAs, dysfunctional antigen presentation, T-cell exhaustion induced by checkpoint inhibitors (such as PD-1), and immunosuppressive cells in the tumor microenvironment may all result in suppression of the immune response and variable patient responses to vaccination.

Therapeutic cancer vaccines utilize a variety of approaches to induce immune activation (Figure 3), including the injection of: whole cell or tumor cell lysates, peptide antigens, plasmid DNA, or activated immune cells primed with tumor antigens [69]. Anti-tumor immunization relies on the presence of TAAs that can be effectively presented to and recognized by cytotoxic T cells and antibody producing B cells [69,70]. TAAs may arise from mutated self-antigens or normal cellular antigens that are overexpressed or abnormally expressed in cancer cells [69]. The success of a cancer vaccine relies on its ability to overcome T cell tolerance to these antigens in order to induce a robust, durable immune response [4].

Currently, one human cancer vaccine, Provenge (Sipuleucel-T, Dendreon Corporation, Seattle, WA, USA), has received FDA approval for use in metastatic prostate cancer [71]. Provenge is an autologous cellular vaccine targeting the prostatic acid phosphatase (PAP) antigen [71]. Despite successfully stimulating a long-lived immune response in a subset of patients, Provenge only extends median survival time by 4 months [70,71]. Although vaccines have been tested in phase-III clinical trials for almost every type of cancer, no other cancer vaccine has received FDA approval, and at least five trials have failed to reach their designated endpoints in the last two years [70]. These trials demonstrate that patient responses to vaccination are highly variable and depend on the ability of the immune system to respond to stimulation [70]. However, most cancers display immune system dysfunction, which includes deficits in antigen presentation, exhaustion of T cells, and an immunosuppressive tumor microenvironment [4,70]. The current challenge in cancer vaccination lies in understanding and overcoming immune system dysfunction, either through improved vaccination strategies or by combining vaccination with other treatment modalities [70]. Veterinary clinical trials represent a valuable opportunity to study the response of the immune system to vaccination and to develop improved vaccination protocols [69].

The first therapeutic cancer vaccine to be approved for any species was the xenogeneic DNA vaccine, Oncept (Merial Limited, Duluth, GA, USA), which was approved by the USDA for use in canine oral melanoma in 2007 [69,72,73,74]. The plasmid cDNA insert encodes for and results in the production of human tyrosinase following intramuscular or transdermal injection [72,73,74]. The human tyrosinase protein is 85% homologous to the canine protein; theoretically, this difference is great enough to break immune tolerance while remaining similar enough to direct the immune response against canine melanoma [74,75]. Conditional approval was based on a study of 9 dogs with stage II to IV oral malignant melanoma (OMM) that demonstrated safety [72]. A two- to four-fold increase in tyrosinase-specific antibodies was observed in three of the nine dogs, and this antibody response was associated with prolonged tumor control [76]. Full approval followed a subsequent study in which the Oncept vaccine was administered to 58 dogs with stage II-III OMM that had achieved loco-regional disease control following conventional surgery and/or radiation [74]. Historical controls were derived from two previous clinical trials in which dogs were treated with surgery and/or radiation alone. The median survival time of the vaccinated dogs was not reached by the end of the study; however, the 25th percentile for vaccinates was 464 days as compared to 156 days for the historical control, which lead the authors to conclude that survival time was significantly improved for dogs receiving Oncept [74]. The efficacy of Oncept, however, does not appear to be universal. A retrospective study that examined the efficacy of adjunctive treatment with Oncept in dogs with stage I to III OMM following loco-regional disease control found no difference in progression free survival, median survival time, or disease free interval between 22 dogs receiving Oncept and 23 stage matched controls, leading the authors to conclude that Oncept provided no therapeutic benefit [75]. This study raised concerns about the strength of evidence used to support both the positive and negative findings in these studies. Both the Grosenbaugh and Ottnod studies used relative small numbers of non-uniform cases that were neither randomly assigned nor blindly evaluated, which increases the potential for biased data [77]. In light of the conflicting data, a prospective, randomized controlled trial is needed to draw conclusions regarding the efficacy of Oncept.

Another genetic vaccine that has been pursued in canine clinical trials encodes a catalytically inactive form of dog telomerase reverse transcriptase (dTERT) [78,79,80]. TERT is the catalytic protein component of telomerase, an enzyme overexpressed in most tumor types to confer immortality to cancer cells [78]. As this protein is expressed in very low levels in normal cells, it is a suitable target for immunotherapy [78]. Two recently published studies have examined the use of dTERT vaccines as adjuvants to standard chemotherapy in dogs with stage III-IV B cell lymphoma [78,80]. In order to induce a robust immune response, the authors utilized two heterologous modes of immunization: electroporation with a DNA plasmid vaccine and administration of a replication-deficient adenovirus vector expressing dTERT [78,80]. The first phase-I trial established the safety of this therapy and demonstrated an increase in overall survival in the vaccine-treated cohort over historical controls (98 weeks *vs.* 37 weeks, respectively) [78]. Vaccination induced a durable, dTERT-specific immune response in 13 of the 14 dogs treated [78]. In a subsequent, double-armed clinical trial, vaccination failed to increase progression-free survival time but did significantly improve overall survival as compared to dogs treated with chemotherapy alone (76 weeks *vs.* 29 weeks, respectively) [80]. Overall, this vaccine appears to be safe in B cell lymphoma.

Since the development of Oncept, a variety of other therapeutic vaccines have been tested in veterinary clinical trials. Autologous tumor cell lysate vaccines supplemented with an immune adjuvant have used in the treatment of meningioma-bearing dogs [81]. Of the 11 dogs vaccinated, all developed polyclonal tumor-reactive antibody responses and infiltration of antibody-producing plasma cells into the tumor. The median survival time of the vaccinated group was prolonged as compared to historical controls (645 *vs.* 222 days, respectively), demonstrating the potential efficacy of this therapy [81]. Vaccination with autologous immune cells, which have been activated and exposed to tumor antigens *ex vivo*, has also been utilized in the treatment of melanoma and B cell lymphoma [82,83]. In a recent clinical trial, activated B cells infused with autologous tumor RNA were administered to 19 dogs with Non-Hodgkin’s lymphoma as an adjuvant to chemotherapy. Vaccination stimulated an anti-tumor immune response and improved the rate of durable second remission, although median time to disease progression and overall survival did not differ between the groups [83]. A recent phase-I trial investigating the use of a DNA vaccine targeting the p62 protein in the treatment of canine mammary tumors reported a reduction in tumor volume in six of the seven dogs injected with no significant toxicity [84]. Decreased growth was accompanied by lymphocytic infiltration and tumor encapsulation, demonstrating the ability of this vaccine to induce an immune response [84]. The use of DNA vaccination has also been investigated for the treatment of metastatic melanoma in gray horses [85,86]. Following an initial phase-I trial [85], a randomized, double blind study was performed in which 26 gray horses with metastatic melanoma were treated with IL-18 encoding plasmid DNA, IL-12 encoding plasmid DNA, or empty plasmid DNA injected intratumorally [86]. Both treatment groups elicited approximately 20% reduction in tumor volume with inflammatory infiltrates seen in 7 of the 10 treated tumors [86]. A number of other DNA plasmid vaccines have been investigated for use in a variety canine, feline, and equine malignancies, as reviewed by Glikin and Finocchiaro (see ref. [87]). The majority of these phase-I clinical trials demonstrated modest efficacy with no significant adverse events.

Although cancer vaccine trials have had more failures than successes, the responses seen in a subset of patients demonstrate the potential to induce an anti-tumor immune response under the right circumstances. Vaccination is most likely to be successful in solid tumors with a high mutation rate (such as melanoma [88]) where a targetable tumor associated antigen can be identified. Improved vaccination strategies or a combination of vaccination with other treatment modalities may be needed to overcome immune tolerance and provide therapeutic benefit for a maximum number of patients. Due to the high degree of variability in patient responses, large, randomized, placebo-controlled studies should be performed prior to regulatory approval.

## 7. Adoptive T Cell Transfer

**Figure 4:**
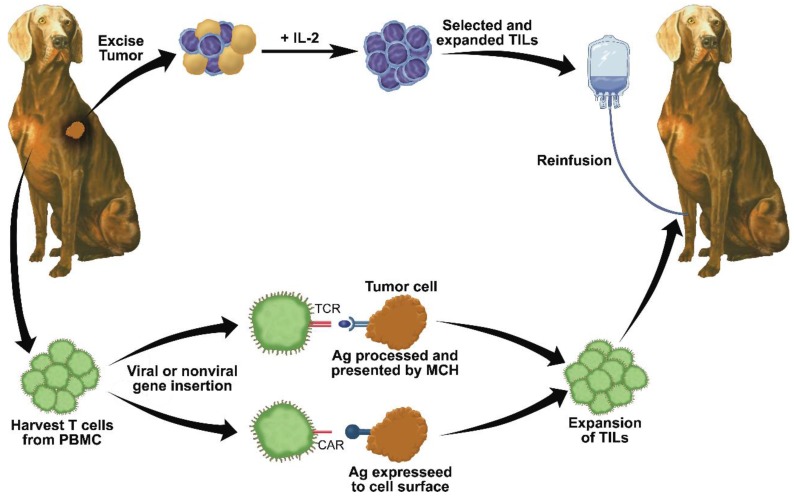
Approaches to adoptive T cell therapy. In the top scheme, a resected tumor sample is digested into a single cell suspension and cultured in the presence of IL-2 to select for naturally occurring tumor-infiltrating lymphocytes (TILs). The TILs are then expanded, tested for anti-tumor activity, and reinfused into the patient. In the bottom scheme, autologous T cells are harvested from the blood and either a transgenic T cell receptor (TCR) or a chimeric antigen receptor (CAR) is introduced by viral or non-viral transduction. TCRs are capable of recognizing a specific tumor antigen presented in the context of an MHC molecule. CARs are MHC-independent and capable of directly recognizing an antigen on the tumor cell surface. Following transduction, the transgenic T cells are expanded and reinfused.

The majority of immunotherapies rely on the activation and expansion of anti-tumor immune cells *in vivo*, which can be impeded by the presence of a profoundly immunosuppressive tumor microenvironment [89,90,91]. In adoptive cell therapy (ACT), autologous T cells are expanded and activated or modified *ex vivo* before being re-infused into the patient, thus circumventing tumor-induced immunosuppression [89,90,91]. These “living drugs” retain their ability to proliferate *in vivo* and to mediate anti-tumor responses [90,91]. Currently, three forms of ACT are being developed for clinical use (Figure 4): tumor-infiltrating lymphocyte (TIL) therapy, T cell receptor (TCR) engineered T cells, and chimeric antigen receptor (CAR) T cells [91].

Tumor-infiltrating lymphocyte (TIL) therapy seeks to isolate and expand populations of lymphocytes with natural anti-tumor activity [90,91]. In this therapy, a section of a patient’s tumor is excised and grown in culture in the presence of IL-2 to promote the selection of lymphocytes, which overgrow and destroy the tumor cells within 2–3 weeks [90]. The TILs are then tested for antitumor reactivity before being expanded *in vitro*. After five to six weeks, up to 10^11^ lymphocytes, typically a mixture of CD8^+^ and CD4^+^ T cells, are infused back into the cancer patient [90]. TILs represent a polyclonal T cell population that may be capable of recognizing multiple tumor antigens [90]. Although TILs can be produced from virtually all solid tumors, only TILs isolated from melanomas consistently demonstrate anti-tumor activity in human trials [90]. It has been suggested that the high rate of mutation seen in melanoma produces neoantigens which can be successfully targeted by the TILs that mediate tumor regression [90]. As dogs possess a fully intact immune system and genetic similarity to humans, TIL therapy in dogs has the potential to inform human trials and investigate the use of this therapy in other cancers, such as hematopoietic malignancies. In a recent clinical trial by O’Connor *et al.*, non-specific, autologous T cells isolated from dogs with non-Hodgkin’s lymphoma were propagated *ex vivo* using a novel artificial antigen presenting cell system prior to reinfusion [5,92]. The infused T cells persisted for greater than 49 days and trafficked to secondary lymphoid organs, demonstrating that the adoptive transfer of autologous T cells is safe in canine patients.

As demonstrated by the failure of TILs to successfully target solid tumors with a low mutation rates, TIL therapy relies on the intrinsic capacity of T cells to recognize tumor antigens [90,91]. To target these tumors, gene-transfer techniques were developed to introduce artificial anti-tumor receptors into normal T cells harvested from the patient’s peripheral blood [90,91]. TCR engineered T cells, expressing one α and one β chain, are designed to recognize known tumor antigens expressed in the context of a MHC molecule [90]. A diverse array of TCRs have been developed and tested in human clinical trials with response rates ranging from 13%–30% [5]. Chimeric antigen receptors (CARs) are artificial receptors composed of a single-chain antibody variable fragment that is specific for a tumor antigen linked to an intracellular signaling domain and co-stimulatory molecules [89,90]. CARs are non-MHC class restricted; therefore, they do not rely on the ability of the patient’s APCs to present antigen and do not need to be syngeneic to the patient’s immune system [90]. Canine T cells expressing a HER2-specific CAR have been developed and display anti-tumor activity against HER2+ canine osteosarcoma cells *in vitro* [93]. Ongoing efforts seek to develop canine CARs targeting B cell lymphoma and other tumors.

ACT holds the potential to induce complete, durable remissions in patients with advanced metastatic disease. To allow the widespread application of ACT in veterinary medicine, new culture techniques need to be developed to reduce the time required for production and the associated costs [91]. Another major challenge facing the development of these therapies is the identification of shared tumor antigens that are not expressed on normal tissues. Unexpected, deadly toxicities have resulted from off-tumor targeting of antigens expressed on vital organs [90]. New approaches seek to utilize whole exome sequencing to identify non-synonymous cancer mutations in order to better understand tumor-lymphocyte interactions and improve targeted cancer immunotherapies [90]. Researchers are also seeking to improve the design of transduction vectors. This includes incorporating genes to enhance T cell trafficking to the tumor site, genes to improve T cell function or reduce microenvironmental immunosuppression, and suicide genes to reverse toxicity [90,91]. Canine trials have the ability to assist in these efforts to optimize adoptive cell therapy for a variety of malignancies.

## 8. Adoptive Natural Killer Cell Transfer

In addition to the recent advances in T cell ACT, the adoptive transfer of autologous and allogeneic natural killer (NK) cells with anti-tumor activity has been investigated [36]. Initial studies utilizing autologous NK cells activated *ex vivo* with IL-2 showed limited efficacy, likely due to inhibitory signals delivered by self-MHC molecules [36]. This lead to the use of allogeneic NK cell transfer, which resulted in prolonged disease free survival rates and several complete remissions in patients with AML [36]. Several additional trials have investigated the use of allogeneic NK ACT in the treatment of solid tumors, but a lack of consistent NK cell expansion limited clinical efficacy and demonstrated the need for a better understanding of NK cell biology as well as improved strategies to overcome immunosuppression [36]. The characterization of canine NK cells [94,95] and the development of culture systems which optimally enhance canine NK cell proliferation and effector function [95,96] allow the investigation of NK cell anti-tumor properties in a veterinary setting. Canine recombinant IL-15, a cytokine that plays a pivotal role in NK cell development and activation, has also been generated [96]. When administered intravenously, rcIL-15 significantly increased the numbers of circulating lymphocytes in the peripheral blood of healthy dogs for up to 11 days after injection [96], indicating that this cytokine could be used to support canine NK cells following adoptive transfer.

## 9. Conclusions

As this review demonstrates, a wide variety of therapeutic modalities have been developed in an effort to activate an anti-tumor adaptive immune response. In addition to the modalities mentioned here, the complex cytokine networks in the tumor environment can be modulated to improve anti-tumor immunity, and these therapeutics have been extensively reviewed elsewhere (ref. [97]). To date, the majority of veterinary immunotherapies have demonstrated efficacy in small, phase-I clinical trials and larger, randomized trials are needed prior to widespread implementation. Although anti-cancer vaccination has progressed farthest in the regulatory pipeline, the variable clinical outcomes seen in response to vaccination demonstrate the need for further optimization of this therapy. Cost and feasibility must also be taken into consideration when developing veterinary therapeutics, making “off-the-shelf” therapies, such as mAbs, AdFasL, and vaccination, reasonable candidates for clinical success as compared to individualized therapies, such as adoptive transfer of TILs. In addition, therapeutic agents utilizing attenuated pathogens, such as oncolytic virotherapy and attenuated bacteria, may need to overcome additional regulatory hurdles prior to approval.

Although the concept of immunotherapy was developed over a century ago, the widespread use of immunotherapeutic agents in the clinic is a relatively recent development, and several obstacles remain to be overcome. The correct dosage, timing, and route of administration of many of the currently used therapies still need to be optimized [3]. Tumors commonly develop resistance to therapy by down regulating recognized antigens or MHC molecules or altering signaling pathways [4]. Therefore, immunotherapy will likely be used in combination with more traditional cancer treatments, such as chemotherapy and radiation [69]. Although conventionally thought to be detrimental to the immune response, recent reports suggest radiation and chemotherapy can induce immunogenic cell death, enhance T cell effector function, and deplete immunosuppressive cells in the tumor microenvironment [98,99,100,101]. These results suggest that, when used with the correct dosing schedule, combination therapies may synergize to promote anti-tumor immunity.

An additional consideration is the potential toxicity associated with the immunogenicity of these reagents. Like conventional cytotoxic chemotherapy drugs and targeted agents, some immunotherapeutics have been associated with severe systemic side effects [30,43,90,102]. These immune-related adverse events, such as severe colitis, hepatitis, pneumonitis, neurotoxicity, and cardiac toxicity, are often manageable with immunosuppressive drugs, but study-related deaths have occurred [30,90]. It is important to note that most immunotherapies have been clinically tested in patients with advanced disease and a long history of therapeutic failures, and who may be at increased risk for systemic toxicity. Toxicities also appear to aggravated in older, obese animals and humans [103]; therefore, young, healthy mice that are often used in preclinical trials are poor models for the human condition. Studies indicate that dogs experience “cytokine storms” similar to those seen in human patients and may be an appropriate model to develop strategies to mitigate toxicity [104].

Immunotherapy is also challenging the way oncologists traditionally evaluate the efficacy of cancer therapeutics. Standard modalities, such as chemotherapy and radiation, have a rapid onset of action and cause a reduction in tumor volume by directly targeting and killing cancer cells. In contrast, responses to immunotherapy may take up to several months due to the time needed to induce an adaptive immune response [3,105]. During that period of time, an increase in tumor size may occur due to the infiltration of immune cells into the mass [3,105]. The unique biological response to immunotherapy has led to the development of immune-related response criteria to evaluate human clinical trials [105], and the implementation of similar criteria may be necessary for the evaluation of veterinary trials.

Despite the remaining challenges, immunotherapy has the potential to revolutionize the field of veterinary oncology. These agents have demonstrated the potential to enact significant, sometimes remarkable, clinical responses in a subset of patients. The challenge now is to understand and optimize these responses to improve the efficacy of immunotherapeutics. Veterinary clinical trials have the ability to not only improve the lives of our patients, but to uniquely inform human clinical trials. With increasing understanding of immune- and tumor-cell biology, immunotherapy represents a pathway to durable responses in our veterinary cancer patients.
